# Lack of focus on nutrition and documentation in nursing homes, home care- and home nursing: the self-perceived views of the primary care workforce

**DOI:** 10.1186/s12913-019-4450-1

**Published:** 2019-09-06

**Authors:** S. J. Håkonsen, P. U. Pedersen, A. Bygholm, C. N. Thisted, M. Bjerrum

**Affiliations:** 1 Centre of Clinical Guidelines – Danish National Clearing house, Department of Health Science and Technology, University of Aalborg, Aalborg, Denmark; 2 Danish Centre of Systematic Reviews: A Joanna Briggs Institute Centre of Excellence, Aalborg, Denmark; 3 Department of Communication and Psychology, University of Aalborg, Aalborg, Denmark; 4 Department of Public Health, Section of Nursing Science, Aarhus University, Aarhus, Denmark

**Keywords:** Focus group, Content analysis, Nutrition, Documentation, Nursing home, Home care, Home nursing

## Abstract

**Background:**

Malnutrition is a comprehensive challenge for the nursing home, home care- and home nursing sector. Nutritional care and the subsequent documentation are a common and multifaceted healthcare practice that requires that the healthcare professionals possess complex combinations of competencies in order to deliver high-quality care and treatment. The purpose of this study was to investigate how a varied group of healthcare professionals’ perceive their own competencies within nutrition and documentation and how organizational structures influence their daily work and the quality of care provided.

**Methods:**

Two focus groups consisting of 14 healthcare professionals were conducted. The transcribed focus group interviews was analyzed using the qualitative content analysis approach.

**Results:**

Six categories were identified: 1) Lack of uniform and systematic communication affect nutritional care practices 2) Experience-based knowledge among the primary workforce influences daily clinical decisions, 3) Different attitudes towards nutritional care lead to differences in the quality of care 4) Differences in organizational culture affect quality of care, 5) Lack of clear nutritional care responsibilities affect how daily care is performed and 6) Lack of clinical leadership and priorities makes nutritional care invisible**.**

**Conclusions:**

The six categories revealed two explanatory themes: 1) Absent inter- and intra-professional collaboration and communication obstructs optimal clinical decision-making and 2) quality deterioration due to poorly-established nutritional care structure. Overall, the two themes explain that from the healthcare professionals’ point of view, a visible organization that allocates resources as well as prioritizing and articulating the need for daily nutritional care and documentation is a prerequisite for high-quality care and treatment. Furthermore, optimal clinical decision making among the healthcare professionals are compromised by imprecise and unclear language and terminology in the patients’ healthcare records and also a lack of clinical guidelines and standards for collaboration between different healthcare professionals working in nursing homes, home care or home nursing.

The findings of this study are beneficial to support organizations within these settings with strategies focusing on increasing nutritional care and documentation competencies among the healthcare professionals. Furthermore, the results advocate for the daily involvement and support of leaders and managers in articulating and structuring the importance of nutritional care and treatment and the subsequent documentation.

**Electronic supplementary material:**

The online version of this article (10.1186/s12913-019-4450-1) contains supplementary material, which is available to authorized users.

## Background

Healthcare systems worldwide use an evidence-based practice (EBP) approach aiming to provide care and treatment of high quality. In order to make the best clinical decisions in day-to-day patient care, care and treatment must be based on information from various sources, such as rigorous research, clinicians’ expertise and patients’ perspectives and preferences [1] Many countries and international organizations have developed evidence based practice guidelines for nutritional care that can be applied and transferred to areas within the primary healthcare sector [2]. Despite the existence of these evidence based practice guidelines [2], malnutrition, especially undernutrition, and the causes of nutritional-related issues are poorly identified in both nursing homes, home care- and home nursing [3, 4]. The poor identification within of malnutrition within these setting have led to malnutrition rates that range from 40 to 90% [5–7]. Malnutrition results in negative outcomes for patients, caregivers and the healthcare system, including increased morbidity, mortality, increased care needs and hospital readmissions [8, 9]. Nutritional care does not only encompass the basic duty to provide adequate and appropriate food and drinks to patients. It also comprises the consistent and systematic assessment, diagnosis, intervention, monitoring and evaluation of factors that can directly or indirectly influence patients nutritional status [10]. In order for healthcare professionals to deliver high-quality nutritional care, several studies stress that the healthcare professionals competencies, the context in which care is delivered (home care or nursing home), collaboration between different healthcare providers and the organizational approach taken are important influential factors [11–18]. Nutritional care is a common, complex and multifaceted healthcare practice that requires precise communication and coordination among different healthcare providers in order to ensure continuity of care and treatment. Nutritional care and the subsequent documentation therefore require that the healthcare professionals possess complex combinations of nutritional and documentation knowledge, routines and attitudes [3, 19–21]. Lack of nutritional care competencies among healthcare professionals negatively influences patient-outcomes and safety-measures [22, 23]. So, despite being a large part of their daily work assignments and tasks it is problematic that healthcare professionals, regardless of their educational level or skills, typically receive minimal training on nutritional care and treatment, as well as the subsequent documentation thereof [3, 24].

The results from a cross-sectional study in a Danish municipality among collaborative healthcare professionals displayed that the documentation routines and level of nutritional knowledge had noticeable variations and inconsistencies. Between 42.1 and 88.2% of the participants in the study were unfamiliar with the locally recommended nutritional screening tools and 61.4–71.4% knew where and how to document patients nutritional problems, including developing care plans [18]. Variations were discovered across and in between three different groups of health care professionals and across health care settings (home care versus nursing home) [18], hence the conclusion that the skills and competencies to practice nutritional care are challenged within these specific contexts.

In order for organizations to implement strategies aiming at increasing nutritional care competencies among their workforce, studies have suggested that research examining the specific competencies of primary health professionals in providing nutrition care and documentation, and the factors associated with delivering a safe and effective care and treatment are conducted [19, 20]. In 2016 a project aiming to map healthcare professionals´ level of knowledge, routines and attitudes towards nutrition and documentation within nursing homes and home care/home nursing was launched. The present study, part of this project, explore some of the questions raised in the first study in the project, the cross-sectional study [18] as it raised a number of questions about possible causal links within nutritional care and documentation.

Firstly; when managers do not consider documentation important enough to give it priority by requesting it as a necessity in the organization, this might have a negative impact on the healthcare professionals’ daily clinical decisions. Secondly; inadequate competencies among the healthcare professionals to perform goal-oriented nutritional care could be an obstacle to high-quality nutritional care and documentation. These questions are explored more thoroughly in the present qualitative study to gain a more detailed understanding of the issues and associations outlined in the survey.

Studies have previously investigated nurses, nursing aids and physicians’ level of knowledge, their practices and their attitudes towards nutrition [25, 26], and other studies have examined documentation routines among different healthcare professionals [27]. The present study is unique in that it is the first qualitative study to investigate nutrition and documentation within a collaborative frame and dynamic, as it examines three different groups of collaborative healthcare professionals, registered nurses, social and health service assistants and social and health service helpers and their self-perceived knowledge, routines and attitudes towards nutrition, documentation, as well as their perceptions of factors that influence their daily work and quality of care provided. The purpose of this study is to investigate how healthcare professionals’ self-perceived views on competencies within nutrition and documentation and organizational structures influence their daily work and the quality of care provided within the nursing home, home care- and home nursing setting.

## Methods

### Setting

The study was conducted in a Danish municipality (population > 70.000) that employs 1134 Social and Health Service Helpers (SSH), 143 Social and Health Service Assistants (SSA) and 120 Registered Nurses (RN). The municipality is divided into four districts with local managements referring to an overall management within nursing homes, home care and home nursing.

### Sampling

The sampling of the participants was carried out by a local coordinator working in the municipality and was based on a convenient sample. This implied that the local coordinator selected those employees fulfilling not only the inclusion criteria’s but also who she assessed would provide the study with the best information. Inclusion criteria matched the workforce within nursing homes and home care/home nursing with maximum variation concerning the following:

The two focus groups were composed of a mix of the inclusion criteria in order to obtain a true reflection of the clinical reality and to enhance discussion.

#### Participants

Seven health care professionals participated in each focus group giving a total of 14 healthcare professionals. Their length of education ranged from 1 yr and 2 months (SSH), to 1 yr and 8 months (SSA) to 3 yrs and 6 months (RN) within the three groups of healthcare professionals. The theoretical part of the SSH and SSA education comprises app. 30–40% of the total. The RN education consists of 60% theoretical education. The practical and clinical training parts of the SSH and SSA education consists of 60–70% of the total whereas the RN education consists of 40% practical training. Table 1 depicts the professional characteristics of the participants.

**Table 1:** Professional characteristics of the participants

	Profession	Place of work (nursing home, home care, home nursing)	Number of years educated (range)	Years of working in nursing homes and/or home care/home nursing (range)
Focus group 1	Registered nurse (1A) Registered nurse (1B) Social and health service assistant (1C)	Home nursing Home nursing Home care	(18 months – 15 years)	(18 months – 14 years)
	Social and health service assistant (1D)	Nursing home		
	Social and health service assistant (1E)	Home care		
	Social and health service helper (1F)	Nursing home		
	Social and health service helper (1G)	Home care		
Focus group 2	Registered nurse (2A) Registered nurse (2B) Social and health service assistant (2C)	Home nursing Home nursing Nursing home	(18 months – 35 years)	(1 year – 31 years)
	Social and health service assistant (2D)	Nursing home		
	Social and health service assistant (2E)	Home care		
	Social and health service helper (2F)	Home care		
	Social and health service helper (2G)	Home care		

#### Data collection

Data was collected using focus group interviews in order to capture the collaborative interactions among the healthcare professionals included [28, 29]. The focus groups were composed of people with similar characteristics as they all were employed within the same municipality, had different educations and collaborated on a daily basis (see inclusion criteria in Table 2).

**Table 2:** Inclusion criteria

Education: - Registered nurses - Social and healthcare assistants - Social and healthcare helpers	
Number of years of education: - Maximum variation of years since completion of education	
Number of years in a primary health care setting: - Maximum variation of years of employment in a primary health care setting (home care, home nursing or nursing homes)	
Employment: - Current employment and working in the municipality was a main criterion	

It thereby provides authentic insights into a cultural collaborative group, through direct access to their interactions, their language and dynamics. Seven healthcare professionals participated in focus group one and seven healthcare professionals participated in focus group two, for a total of 14 healthcare professionals. In focus group one, the years of working within these specific settings varied from 18 months – 14 yrs. In focus group two, the years of working within nursing homes and/or home care/home nursing varied from 1 yr to 31 yrs.

The two focus groups interviews were conducted by SJH who is an experienced registered nurse and MB who is an experienced qualitative methodology researcher. MB primarily attended the focus groups as an observer ensuring that ethics and all interview aspects were addressed. The focus groups interviews were conducted in September 2017 and lasted 84–94 min and took place in a secluded and private meeting room, without the disturbance of colleagues or managers. The discussions among the focus groups participants were audiotaped and transcribed verbatim including non-verbal signs such as laughter and hesitating by a transcription service and carefully checked for transcription errors and accuracy by (SJH).

A semi-structured interview guide was used to steer the focus groups towards the phenomena of interest and to ensure consistency. The interview guide have not been published elsewhere (see Additional file 1). In order to ensure internal validity the interview guide was designed to respond to the nine assumptions revealed from the survey study [18], see Table 3.

**Table 3:** Overview of the study’s validity: From assumptions to questions

Assumptions	Purpose	Questions
*Routines in relation to nutrition:* Healthcare professionals are not well-trained or educated in identifying nutritional problems, setting up goals, identifying interventions and evaluating nursing sensitive outcomes.	To investigate how healthcare professionals’ self-perceived views on competencies within nutrition and documentation and organizational structures influence their daily work and the quality of care provided within the nursing home and home care/home nursing setting.	-How do healthcare professionals describe their specific nutritional routines in their daily work?
*Routines in relation to documentation:* Healthcare professionals are not well-trained or educated in systematically developing care plans		-How do healthcare professionals describe their specific documentation practices in their daily work?
*Knowledge in relation to nutrition:* Malnutrition or nutritional issues are often overlooked in nursing homes and home care/home nursing, because the healthcare professionals lack awareness and knowledge of which variables affect and influence patients’ nutritional state, hence affecting the quality of care.		- Do the different healthcare professionals consider that their education sufficiently prepares them to provide nutritional care?
*Knowledge in relation to documentation:* Healthcare professionals are not using existing nutritional screening tools or guidelines because they are not aware of how to put them to use in a clinical setting.		- Do the different healthcare professionals consider that their education sufficiently prepares them to document and develop nutritional care plans?
*Attitudes towards nutrition:* Malnutrition or nutritional issues are often overlooked, because healthcare professionals do not prioritize nutritional care.		-Do healthcare professionals consider nutrition to be an important part of their job and daily tasks?
*Attitudes towards documentation:* Malnutrition or nutritional issues are often overlooked, because healthcare professionals do not prioritize documentation.		-Do healthcare professionals feel that documentation is an important part of their job and daily tasks?
*Factors that affect daily work and quality of care (organizational obstacles):* Nutrition and documentation routines are not specified and clear for the healthcare professionals and could therefore have a negative impact on both the daily workflow and continuity of care and treatment. Healthcare professionals are not using existing screening tools or guidelines because they do not feel obliged and are not required to do so		-Does their place of work have clear guidelines for routines regarding nutrition and documentation within and between the three groups of healthcare professionals? -How do the different healthcare professionals’ experience enabling the retrieval and use of nutritional screening tools?
*Factors that affect daily work and quality of care (context/setting):* The performance and execution of nutritional related activities could be influenced by the fact that observations and interventions are done in the patients’ home, lacking, for instance, a weighing scale.		-Do healthcare professionals consider the context of their workplace as an obstacle? -Does their workplace prioritize nutrition and documentation in their daily work?
*Factors that affect daily work and quality of care (collaboration between different healthcare providers):* Malnutrition or nutritional issues are often overlooked, because healthcare professionals are not aware of their specific role and collaboration with other caregivers in nutritional care and subsequent documentation. Variation in healthcare professionals’ nutritional routines and documentation practices could influence both the workflow and continuity of care and treatment.		-How do healthcare professionals with various educational levels describe and consider their nutritional care responsibilities? -How do healthcare professionals with various educational levels talk, describe and consider their responsibilities regarding documentation? -Do they describe or outline any ambiguities or disagreements in terms of their routines and practices?

The interview guide comprise six domains: 1) Routines in relation to nutrition and documentation, 2) Knowledge in relation to nutrition and documentation, 3) Attitudes towards nutrition and documentation, 4) The context of their daily work, 5) Collaboration between different healthcare professionals and 6) The organization of their employment. Examples of questions are shown in Table 4. Each domain of the interview guide consisted of several questions (between 4 and 12 questions within each domain) and probing questions which were used to explore and clarify the participants views were used to assist and support SJH and MB in the focus group situations if the conversations and discussions among the participants were not running smoothly or there were confusion or insecurity related to the questions asked.

**Table 4:** Example of questions in the interview guide

Domain	Examples of interview questions
Routines (nutrition and documentation)	- Is nutritional care a routine task in your workplace? - What are your specific daily tasks or routines in relation to nutrition? - Do you experience that your daily routines are consistent? - Do you develop nutritional care plans? - When do you think that it is necessary to develop nutrition care plans? - Can you tell me how you work with and use documentation in your daily work? - Do you consider a primary care context as an advantage or disadvantage when planning and documenting nutritional care? - How do you primarily communicate nutritional related observations with your colleagues?
Knowledge (nutrition and documentation)	- Can you tell me which type of knowledge you base your nutritional advice upon? (evidence /expert)? - Can you mention some of the latest nutritional advice you gave a patient? - Do you feel that you know enough about nutrition? - Where do you seek guidance concerning nutritional care or documentation if needed? - How do you become aware of nutritional issues with the patient? - What do you do if a patient is malnourished or at risk of malnutrition? - Do you know of existing nutritional screening instruments? - Do you know how to develop nutritional care plans?
Attitudes (nutrition and documentation)	- Do you consider nutrition to be part of your responsibility? - Do you all have the same degree of responsibility or are there different levels of responsibility? - Is nutritional care important? Does it “work”? - Do you think that documentation supports you in your daily work? - Is documentation a priority in your workplace?

#### Data analysis

The transcribed interviews were analyzed according to the qualitative inductive content analysis methodology [30–32] and ensuring validity focused on how the manifest and latent content of the informants’ views explain the described assumptions [33–35]. The participants’ views and perceptions were constantly analyzed and considered within the social interaction dynamics. All observations on group dynamics were written down during the focus groups and were subsequently analyzed and assessed within the context of their collaborative interaction. No social interactions dynamics theory was however included in the analysis, as the observations on the participants interactions were analyzed within the content analysis frame. Consensus, disagreements and diverse views among the informants were acknowledged and emphasized as equally important by the interviewers.

**Fig. 1:**
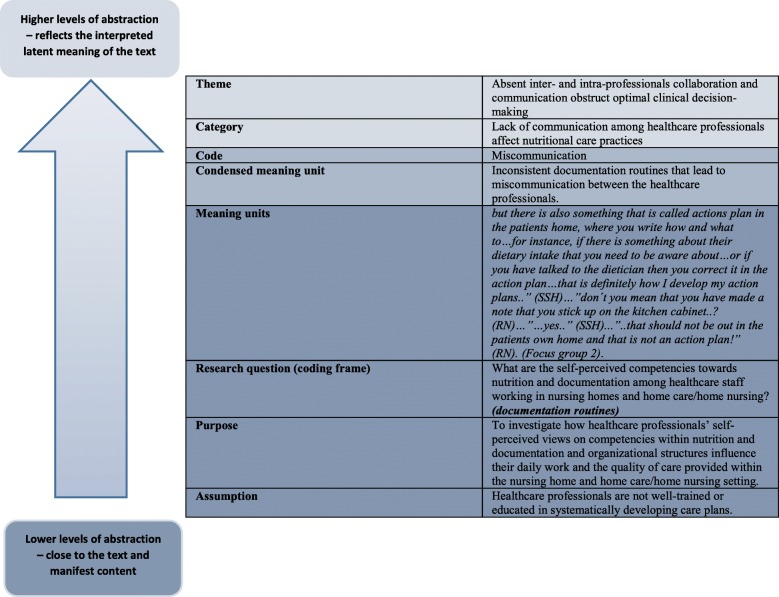
Example of the analytical process (figure by Erlingsson et al. 2017 [37])

The analysis was conducted in four steps. Firstly; the interviews were read by SJH several times to gain an overall understanding of the transcripts and notes were made throughout the reading. To increase reliability the reading started at different pages each time [36]. Secondly; meaning units relevant to the purpose of the study was identified using two research questions: 1) What are the self-perceived competencies (routines, knowledge and attitude) regarding nutrition and documentation among registered nurses, social and health service assistants and social and health service helpers working in nursing homes or home care or home nursing? 2) Which factors (context, collaboration, and organization) do registered nurses, social and health service assistants and social and health service helpers believe influence their daily work and the quality of care provided? Thirdly; (the descriptive level), the derived meaning units were labelled and coded which described the condensed meaning units. The codes were then examined for similarities and grouped together into six categories, hence describing the essence of the healthcare professionals self-perceived knowledge, routines and attitudes towards nutrition and documentation and the quality of care delivered. Fourthly; (the explanatory level), these categories were comparatively examined to interpret and explain how healthcare professionals perceive their own competencies as well as the organizational structures and finally compromised to two overall themes [32]. The analysis was conducted in a constant dialogue between SJH and MB, and the main outlines were discussed with PUP and CNT in order to rule out misunderstandings and maximize validity. An example of the analysis process is shown in Fig. 1.

To increase the validity of the study, an inter-rater reliability test was performed. An inter-rater reliability test examines the extent to which two or more independent coders obtain the same result when using the same coding frame [38–40]. SJH and CNT both familiar with qualitative content analysis methodology coded part of the transcripts [41]. Prior to the coding process SJH carefully introduced CNT to the coding frame. From a sample of the transcripts SJH and CNT independently extracted meaning units using the two research questions. The coded meaning units were then compared and reasons for disagreements discussed and a refined set of meaning units were agreed upon [41]. After the categories were developed, SJH coded the meaning units from the all transcribed interviews to the list of categories. CNT then independently attached these categories to segments from a selected sample of the transcribed interviews [38–40]. The two coders then compared the set of codes that each other had assigned to the text and discussed their reasons for their disagreements and refined the categories and codes. Coding and statistical analyses were made by using ReCal2: Reliability for 2 Coders.

The inter-rater agreements were calculated for both research questions and meaning units (Krippendorfs Alpha 0.79) and meaning units and categories (Krippendorfs Alpha 0.85) adopting Krippendorff’s alpha reliability coefficient ranging from 0 (complete disagreement) and 1 (complete agreement) [38–40]. No cut-off for an acceptable Alpha was established beforehand, as it was the degree agreement and disagreement that determined the final Alpha, which was therefore used to clarify and focus the analysis process.

An Alpha of 0.79 and 0.85 was therefore accepted as the disagreements between the two coders only were related to the length of extracted meaning units and not related to the overall content or meaning.

## Results

The analysis of the data material collected from the two focus groups that consisted of 14 healthcare professionals views and experiences on nutrition and documentation in nursing homes and home care/home nursing revealed six categories: 1) Lack of uniform and systematic communication affect nutritional care practices 2) Experience-based knowledge among the primary workforce influences daily clinical decisions, 3) Different attitudes towards nutritional care lead to differences in the quality of care 4) Differences in organizational culture affect quality of care, 5) Lack of clear nutritional care responsibilities affect how daily care is performed and 6) Lack of clinical leadership and priorities makes nutritional care invisible**.**

### Lack of uniform and systematic communication affect nutritional care practices

A lack of recognition of the benefits of documentation and inaccurate communication resulted in inconsistent and random routines concerning nutritional care which affected continuity of care and treatment.

Communication encompasses both what is reported in the patients’ healthcare record and also what is verbally articulated and the terminologies used. Primary health care faces the challenge of imprecise clinical language within and across the groups of healthcare professionals. Basic clinical terms such as “action plans” or “care plans” are misunderstood and applied by the healthcare professionals in different ways, leading to a miscommunication related to multiple terminologies which affects their daily practices;

*“:..but there is also something that is called actions plan in the patients home, where you write how and what to…for instance, if there is something about their dietary intake that you need to be aware about…or if you have talked to the dietician then you correct it in the action plan…that is definitely how I develop my action plans..” (SSH 2F)…“don’t you mean that you have made a note that you stick up on the kitchen cabinet..?” (RN 2A)…“…yes..” (SSH 2F)...“..that should not be out in the patients’ own home and that is NOT an action plan!” (RN 2A). (Focus group 2)*.

Documentation is not considered to be the primary mean to communicate among the healthcare professionals. This compromises the continuity of care. Furthermore, the benefits of daily documentation are purely related to legal issues and are not described as beneficial in the day-to-day delivery of care and treatment within and between groups of healthcare professionals;

“:. *I think that the overall intention is that we should primarily communicate through the patients’ healthcare record, but…” (SSA 2C)“…doing so, will make the little details and nuances disappear…” (RN 2B) (Focus group 2).“..primarily we are documenting in the patients’ healthcare record, so that if someone comes and say; “I have not had that service”, then you can go in and see that someone in fact has delivered this service..” (SSA 2D)“…I mean, it is to avoid those newspaper front pages..” (RN 2A)(Focus group 2)*.

Healthcare professionals’ lack of acknowledgement of documentation as a useful tool in their daily clinical decision-making display uncertainty regarding how and where to document relevant information, observations and interventions about patients’ nutritional status. This leads to a lack of transparency regarding where data about the patient are documented, and also underlines that routines regarding documentation are random, inconsistent, non-systematic and person dependent;

*“…I am really, really excited to hear where you document it?..” (RN 1A)“…under nutrition. I develop an action plan name it nutrition..” (RN 1B)“…we also do that in x district…” (SSA 1C) (Focus group 1)*.

Inconsistent documentation routines negatively affect the possibility of healthcare professionals acting in a timely and precise manner, and of initiating and continuing relevant interventions;

*“... But when you are out there in the citizens home, as a new employee, you think why has he not eaten anything… is he sick or what is wrong? Is it the teeth or something else? Then it turns out that it was something entirely different…but it wasn’t written anywhere…but then someone said that they had heard something about him...” (SSH 1G) (Focus group 1).*

### Experience-based knowledge among the primary workforce influences daily clinical decisions

Care and treatment in nursing homes, home care- and home nursing are challenged by a lack of applying or demanding different types of evidence within the clinical decision-making process.

There was no formal evidence sharing within the nutritional area among the healthcare professionals. Healthcare professionals do not consistently consider or apply other forms of knowledge than their own and colleagues’ expertise in the clinical decision-making process. Attention to existing guidelines or other sources of evidence was lacking in the daily practice. Healthcare professionals have a one-sided perception of what nutrition is and articulate it as being a form of food service. In addition, their knowledge on nutrition principles was sparse as they have difficulties explaining which actions and interventions are appropriate in some situations, as well as explaining the connection between observations and interventions.

*“…there is always someone who has tried something, right?..” (SSA 1C)“…then you can ask them, what you should do in the particular situations..” (SSA 1D) (Focus group 1).*

*“…but then, when you are out here, then I think that years of experience enable you to know, that you can do so and so and so..” (SSH 1F)“…I ask my colleagues what they usually do…” (SSH 1G) (Focus group 1)*

*“…we do not have a forum where we share knowledge or anything else. It is up to ourselves to know who may know something about particular topics..” (SSA 1E)“… yeah, its learning by doing..” (RN 1A)(Focus group 1)*.

### Different attitudes towards nutritional care leading to differences in the quality of care

Healthcare professionals, regardless of their educational level, articulate nutritional care and documentation as important. Despite this, nutrition and documentation are not given the same priority as other care activities within their daily tasks.

Overall, both nutritional care and documentation are perceived to be important and part of the daily job assignment and the responsibility and delivering of nutritional care and documenting relevant observations is acknowledged. However, the quality in the delivery of care within these areas is inconsistent, as it is interest- and person dependent. This means that if a healthcare professional has a special, or no special interest in nutrition, then the quality of care and treatment of the patients are dependent of this;

*“…but I think that we all are aware of how important it is (nutrition)” (RN 2A)“…but what about the poor patient? The patients who are dependent on the healthcare professional’s interest in nutrition. As a healthcare professional you are the only one who should be able to connect problems with nutrition…” (SSA 2D)“…and then there are some healthcare professionals who do it more than others because they have a higher interest in the topic and prioritize it…” (SSA 2C) (Focus group 2).*

This leads to a varying quality in the delivery of care and treatment, as it is influenced by the individual professional’s preferences and values specifically concerning nutritional care.

Furthermore, there are different attitudes and opinions as to whether the healthcare professionals should spend more time on documenting nutritional care and treatment in patients’ healthcare record. Some healthcare professionals feel that they already spend too much time on what they call useless documentation while other healthcare professionals would like to have more time allocated to documenting. Overall, there was a difference of opinion within the three groups of healthcare professionals, with registered nurses perceiving that documenting nutrition care was time consuming and taking focus away from *“real issues in real life”.* Overall, social and health care assistants and helpers would like to spend more time documenting as they feel they have too little of this in their daily work and regard is important in relation to ensuring continuity of care;

*“…no, I would not spend more time documenting!..” (RN 1A)“..I mean, it is exactly it… we spend, I mean, a lot of time documenting. We have had this new horrible system for 1 ½ year and it just takes so much time… I don’t think anyone is interested in spending more time documenting…” (RN 1B)“…but it is a huge issue in the media, how much time us nurses spend on documenting, so we are not unique in that sense…” (RN 1A)(Focus group 1).*

*“…I actually think I would like to spend more time documenting..” (SSA 2C)“…because there is a lot to document..” (SSH 2F)“…it is really important to document about those things to benefit both the patient and myself. Your colleagues need to know what is going on..” (SSA 2C)“..and it is not always that we have time to even write anything..” (SSH 2F).(Focus group 2).*

### Differences in organizational culture affects quality of care

Although healthcare professionals are employed in the same organization and municipality, there are significant variations in the daily routines and of what is considered an acceptable quality of care and treatment, when employed in home care, home nursing or the nursing home setting. The different quality standards and the quality differences within these two settings are reflected in the care and treatment of patients with the risk of exposing patients to negative outcomes and safety.

Overall, the healthcare professionals working in primary health care consider the primary care context as a challenge in their daily work, hence affecting the quality of care. Working in the patient’s own home poses special challenges. Health care workers can feel intimidated and tend to withdraw into themselves and not question patients’ preferences although this may have a direct negative impact on the patient. It feels difficult to dictate and recommend what patients should do, what to eat and how to act like when in the patient’s own home. Furthermore, healthcare professionals perceive their role in the patient’s home solely as a guest providing service and not a professional healthcare worker with knowledge, skills and valuable insights;

*“…I also feel that you accept a NO to quickly…or what should you say. If you come in to the patients home and should serve the food, and they say “no, I don’t want it, I am not hungry” – then you just accept it, right. Instead of trying to in some way to say something that can encourage the patient to eat their food…” (SSA 1D)“…and it is probably because that you are told that you should accept, erm, the patients’ own wishes or whatever..” (SSA 1C)“…it must also be super annoying that someone comes and say that you should eat your food..” (SSA 1E) (Focus group 1)*.

Furthermore, it is difficult to provide care in patients’ homes, as there is an imbalance in the patient, relative and care provider relationship, as the patient’ inevitable autonomy in their own home may result in health care professionals refraining from performing professional care and treatment. The unequal and imbalanced relationship affects the quality of care delivered, as the healthcare professionals set aside what they have been trained to do in their education regarding providing nutritional care advise and counseling. This is reflected in professional insecurity among the healthcare professionals, as they have to navigate on uncertain path and propose alternative solutions in situations where their professional training is no longer adequate;

*“…but there you definitely face some challenges..” (SSA 1C) “..when facing the patient and their family, then you are just…set aside. Then all of our training is just thrown to the ground..” (SSH 1G)“…because, it is actually what we basically have been taught in our educational training..” (RN 1A)“…so right now, we are actually heading away from what we have learned..” (SSA 1C)“..and do the opposite of what we think should be done..” (RN 1A)(Focus group 1).*

Also, there are differences in the practices and quality standards across settings in the same organization and municipality (home care versus nursing home), which affects the quality of care delivered and may cause quality deterioration, specifically within the homecare and home nursing setting. The daily use of e.g. blood sugar schemes and nutritional screening instruments are associated with large variations. Tools that are useful to apply in nursing homes are not always transferred and used in homecare or home nursing, despite their relevance of use in both settings. The healthcare professionals are aware of these differences and practices, yet they do not speak of them as *quality differences* or *quality deterioration*. Overall, they accept that there are significant differences in practices within different settings;

***Blood sugar schemes:*** *“…when I started working in this municipality I started out in a nursing home. And there I was taught to use these blood sugar schemes. Then I was transferred to home care and was told that I under no circumstances should use these schemes. These were really odd to me, as I actually thought that they were really useful..” (SSA 1E)“.. I don’t understand that. What are the difference between being in a nursing home and home care?” (RN 1B)“…I don’t know – but it should be the same…especially in the same municipality” (SSA 1E) (focus group 1).*

***Nutritional screening instruments:*** *“.. No, we don’t use nutritional screening instruments in the patient’s home. In nursing homes I definitely think they do..” (SSH 2G) “..and in the hospitals. That’s where you screen for nutritional issues, right?..” (SSA 2D)“.. but we have some..” (SSA 2C)“..yeah, they exist..” (SSA 2D)“…sure..” (SSH 2G) (Focus group 2).*

### Lack of clear nutritional care responsibilities affect how daily care is performed

Confusion and insecurity about professional functions and areas of responsibilities within the nutritional area and documentation affect the quality of care delivered.

When patients’ nutritional problems and issues are identified by an employee, it is difficult for the individual employee to figure out who they should contact and refer to in order to gain further help or assistance.

*“..I mean, that’s where the challenge is. It’s not that there aren’t any food service suppliers or something like that.. that’s not the problem. The problem is who you should get a hold of if an issue arises..” (SSA)(Focus group 2).*

The divisions of responsibilities and functions within or across the three groups of healthcare professionals are not clearly defined. It is unclear what each healthcare profession’s responsibility encompasses and when to involve colleagues or contact other healthcare professionals. Regardless of educational level, all health care professionals consider themselves as being responsible for nutrition and the subsequent documentation in the patients’ healthcare record, although they have difficulties being explicit about who does what, when and where.

*“..No, we do not have specific or different levels of responsibilities. We all should do the same..” (SSA)“…I mean, in our district, if we see that someone might have a nutritional problem, we usually visit them and them…er…we contact the nurse or dietician if it is really bad..” (SSA)“..I’m thinking that it primarily is the ones who are with the specific patient, who are responsible, regardless of their education…(RN)” (Focus group 1)*.

### Lack of clinical leadership and priorities makes nutritional care invisible

There is a perceived lack of organizational and clinical leadership on priorities of nutritional care and documentation. They also perceive that there is no systematic approach to training and educating both existing and new employees in documentation systems or in nutritional care guidelines, which results in the healthcare professionals having a varied and inconsistent approach to these areas in their daily work.

Lack of time and lack of resources are perceived to be an important influence on their daily work and quality of care. Furthermore, nutritional care and documentation are not a priority or on the agenda within the primary health care setting.

***Time:*** *“..well, we are not supposed to say it. But lack of time is an important factor that prevents us from doing our job…” (SSH) (Focus groups 1)*

***Agenda:*** *“..no, I don’t think that nutrition is on the agenda here…” (SSA) (Focus group 2).*

Training and education in documentation and nutritional care practices have not been conducted systematically or regularly, as training is typically done by decoding colleagues’ practices. This entails that new employees are trained differently and that training within an organization becomes person-dependent, as there are no guidelines or procedures to follow when new healthcare professionals are hired and must be trained. Overall, this leads to differences and discrepancies in daily routines and practices among the healthcare staff.

*“…it is nothing but a decoding of my colleagues practices..” (SSA)“…I mean, I think that within nursing, whenever a new colleague arrives, then an employee gets the job to train and teach this new colleague. But one employee teaches routines and practices in one way, another in a different way. So there are no rules. No structured training. So two new colleagues can be trained totally differently, because there are not structure for our practices and training…” (RN)(Focus group 1)*.

The differentiated training and education influence the healthcare professionals’ daily routines, as their lack of specific knowledge and insight into e.g. existing nutritional screening tools and electronic support systems causes their daily work to be sub-optimal and inefficient. The is no transparency as to which tools, guidelines or systems should be applied in their daily work;

*“…it’s impossible to be aware of which tools to use, when the training we got from our workplace was so deficient and incorrect..”(SSH)“…plus, there are all other kinds of tools that I talked to our leader about. Then there is this and that tool, which actually are the same as this one. However, we should not use any of them, because they only exist on paper, so it takes extra time to apply it to the documentation system. So that’s a NO-GO…” (RN)(Focus group 1)*.

## Discussion

This study investigated how healthcare professionals’ view their own competencies within nutrition and documentation and how organizational structures influence their daily work and the quality of care provided within the nursing home, home care- and home nursing setting.

The transversal analysis of the categorized meaning units [32] revealed two explanatory themes: 1) Absent inter- and intra-professional collaboration and communication obstructs optimal clinical decision-making and 2) quality deterioration due to poorly established nutritional care structure.

Overall, the two themes explain that from the healthcare professionals’ point of view, a visible organization that allocate resources, prioritizes and articulates the need for daily nutritional care and documentation are a prerequisite for high-quality care and treatment. Furthermore, optimal clinical decision making among the healthcare professionals is compromised by imprecise and unclear language and terminology in the patients’ healthcare records and also a lack of clinical guidelines and standards for collaboration between different healthcare professionals working in nursing homes, home care and home nursing.

### Absent inter- and intra-professional collaboration and communication obstructs optimal clinical decision-making

The collaboration and documentation within and between the different healthcare professions are compromised by poor documentation and moderate professional knowledge of and attitude to nutritional care. Inadequate documentation and knowledge about causes and effects of e.g. change in dietary intake or weight changes among patients in primary health care, may lead to daily clinical decisions regarding care and sub-optional treatment. This is in line with results from a meta-analysis of the evidence of the effect of nutritional knowledge on daily processes and patient outcomes, where it is concluded that it can have severe consequences for the patients when the healthcare professionals’ knowledge or understanding of nutrition are sparse, and it specifically concluded that improvements in the level of knowledge about nutrition had a positive influence on the nutritional related documentation performed by registered nurses. [42]. A significant beneficial effect of increased nutrition knowledge among healthcare workers in nursing homes was found on a range of outcomes, such as increased nutritional intake, improvements in weight and body composition, improvements in reported eating difficulties and finally a significantly lower prevalence of malnutrition in patients [42]. Increased knowledge and understanding about nutrition and documentation can improve nutritional care practice. This emphasizes the need for an organization focus on the establishment of a locally developed post-graduation education for all healthcare professionals involved in both nutritional care and documentation.

The present study identified problems related to imprecise, inconsistent and ambiguous clinical language and terminology. The healthcare professionals had heterogeneous understanding and use of clinical terms, such as action plans and nutritional care, leading to misunderstandings and challenges in their daily routines and practices. Imprecise use of language leads to an inadequate information transfer between different healthcare professions and therefore influences the clinical decision-making process. Specifically this may result in a lack of initiating nutritional related interventions and thereby increasing the risk of adverse patient outcomes, such as malnutrition [43]. The continuity of nutritional care and treatment is complicated by a lack of inter- and intra-professional communication across the municipality because the clinical language and the terminology that the healthcare professionals apply orally and in writing is neither consistent nor understood in the same manner by the healthcare professionals. This is consistent with findings from another study that found that patients with identical symptoms or similar problems might receive different diagnoses, due to the different use and understanding of clinical terms and language [44]. Furthermore, it has been identified that lack of a precise language and consistent terminology makes it impossible for clinicians, researchers and mangers to aggregate, share and reuse data from patients’ healthcare records, as the current definitions and understandings of clinical terms are ambiguous and associated with how the individual healthcare professional perceive the individual terms [45–47]. Therefore, efforts should be made to establish a common terminology and language that are understood and applied in the same way by all healthcare professionals thus ensuring that patients receive better standard of care in daily clinical practice.

In the present study, it was perceived that the healthcare professionals’ prerequisites for delivering high-quality nutritional care are affected by lack of and poorly understood formal guidelines for the daily workflow and collaboration within and in between the different healthcare professions. For instance, the confusion and lack of transparency related to which healthcare professionals and professions are responsible for specific areas within nutritional care and documentation makes it difficult to understand who does what, when and where. Finally, this may lead to interventions being performed twice by different healthcare professionals or that nothing gets initiated or done. Similar challenges have been identified in other studies within different settings [48, 49]. Within hospital settings, with diverse wards, discrepancies in mutual perceptions of collaboration among different healthcare professionals have been identified. The confusion and problems related to the healthcare professionals’ collaboration and interactions leads to problems in the coordination of patient care and treatment essentially influencing the quality of care provided to the patients [48, 49]. Furthermore, research has repeatedly investigated and documented the impact of collaboration problems on both work processes and patient safety and outcomes [50, 51]. For example, failures of collaboration were found to be at the center of a number of care failures, such as communication failure due to missing documentation, poor planning of patient care courses, misdiagnoses of patients due to communication failure and inappropriate admissions or readmissions to hospital [52, 53]. Therefore, professionals must ensure that they collaborate and communicate effectively by the use of consistent terminology in an effective manner to deliver safe, high-quality patient care.

### Quality deterioration due to poor-established nutritional care structure

In the present study it was found that the leaders and managers do not sufficiently prioritize nutritional care and documentation to a high level of quality level, and they do not allocate resources targeted on continuous, systematic training in nutritional care and documentation. This is a problem as studies have shown that such a lack of focus on systematic training and increasing skills and competencies are a barrier for the early identification and treatment of undernourished patients [14, 54, 55]. Studies have reported that the organizational support and prioritization for nutritional care is important in order to achieve improvements in e.g. nutritional therapy and a decrease in malnutrition among patients at risk of malnutrition [54, 55]. A systematic review of randomized controlled trails has investigated the consequences of a missing nutritional care structure specifically within the community setting on both patient outcomes and daily processes [56]. Well-integrated and well-established models and organizations of care have been shown to improve processes of care, for instance documentation and collaboration between healthcare professionals. Furthermore, these well-established structures and models of care have the potential to reduce hospital admissions, readmissions and the use of home care and home nursing services [56]. It is therefore crucial, that an organization requires that local leaders and managers allocate resources for continuous and efficient nutrition and documentation training of its staff in order to support successful change within nutritional care and its subsequent documentation.

The healthcare professionals’ decisions about the point of care are experience based, as their clinical decisions rely on their own and their colleagues experience within the nutritional area and documentation. They do not refer to national guidelines on specific areas of nutritional care, e.g. how to calculate protein and energy intake for the individual patients nor do they refer to locally developed descriptions of standards of care. If clinical decisions are based on evidence, risks of initiating nutritional interventions or actions that may be redundant or even harmful to the patients are lessened [57]. An evidence-based approach requires that the healthcare professionals not only considers their own experience, but also considers knowledge from the best available scientific evidence and the patient’s preferences in the clinical decision-making process [1]. An organization that systematically implements and continuously articulates an evidence-based practice approach has been shown to generate positive improvements concerning its healthcare professionals’ knowledge, their attitudes and daily skills [58]. Therefore, when leaders and managers do not focus on and take explicit responsibility for making other sources of evidence available and useful for the healthcare professionals employed in their area and require that they incorporate it in their daily clinical decisions, it has a negative impact on patients’ nutritional care and treatment.

In the municipality studied, there are substantial variations in the healthcare professionals’ daily routines and practices regarding nutritional care and documentation. The use of different schemes and instruments, such as blood sugar schemes and nutritional screening instruments, are well integrated in some parts of the municipality, especially within the nursing home setting, and are highly beneficial for the patients as they support and facilitate the co-ordination of care and treatment through significantly improved documentation [59]. The use of a validated nutritional screening instrument is associated with better nutritional care and lower malnutrition prevalence rates [59]. It is problematic, that in other parts of the municipality, especially within home care and home nursing, these available schemes and instruments are not consistently integrated and applied, as the initiation of tailored nutritional interventions is lacking. Therefore, the quality differences within the same municipality that have been identified in our study are due to organizational structures that are not consistent in all parts of it. An organization should be attentive to establishing common nutrition and documentation guidelines for patients with identical symptoms and problems, so that it can be expected that all patients, regardless of where they live, will receive high-quality care and treatment.

### Methodological considerations

The strength of this study is the richness of data obtained from the group dynamics in the focus group interviews. The participants in the focus groups represent a realistic staff composition in primary health care with regard to their educational level, their age, their number of years of education and the number of years they had worked in nursing homes and/or home care/home nursing. The maximum variation and representation of a true clinical practice was an advantage, as it allowed different kinds of perspectives to be discussed. However, this could also be considered as a potential disadvantage, due to the inequalities of education, power and hierarchy within the groups (participants with 3 ½ years of education and participants 1 yr and 2 months in the same group). However, there were no obvious signs of imbalance in power or a hierarchy which might have prevented all participants from giving their points of view. Wengraf (2001) [60] and Wilkinson (1998) [61] claim that there is less risk of power imbalances in focus groups than in one-to-one interviews, where the researchers often have more power.

Furthermore, the composition of the themes was not only developed on the basis of the data from the transcribed interviews, but also on the basis of the discussions among the focus group participants. A focus group interview approach was chosen in order to detect and observe the dynamics and the interactions among the three groups of healthcare professionals who on a daily basis collaborate and work together. The observations made by the authors SJH and MB during the interviews supports the developed themes and therefore also validate our findings.

It is a methodological consideration that two of the authors (SJH, MB) were also participants in the focus group discussion; that is, they represent both an insider and an outsider perspective. A possible problem related to insider and outsider perspective was the mixed roles of the authors, as they were both participants in the focus group interviews and also those who interpreted the data. There is a risk of bias due to pre-understandings of the topic and the context. In order to overcome this potential bias, a third author (CNT), who was not involved in the focus group interviews, was involved in different phases of the analysis process (see the section on inter-rater analysis) to increase the validity and reliability of the results.

## Conclusion

This study provides important information regarding the self-perceived competencies and factors that influences daily quality of care among a diverse and collaborating group of healthcare professionals within the nursing home and home care/home nursing setting.

Knowing that adequate nutritional status has an important impact on patients’ physical and psychological well-being, priority needs to be given to safety and quality including an increased focus on healthcare professionals’ competencies within nursing homes and home care/home nursing. If nutritional care and the subsequent documentation are not considered as part of daily treatment and care by the organization and municipality, then it cannot be expected that the healthcare professionals perform high-quality nutritional care.

### Relevance to clinical practice

The findings of this study are beneficial to support organizations within these specific settings with strategies focusing on increasing nutritional care and documentation competencies among their healthcare professionals. Furthermore, the results call for the daily involvement and support of leaders and managers in articulating and structuring the importance of nutritional care and treatment and its subsequent documentation.

## Additional file

Additional file 1: Interview Guide. (DOCX 17 kb)

## Data Availability

The datasets used and/or analyzed during the current study are available from the corresponding author on reasonable request.

## Abbreviations

RN: Registered nurse
SSA: Social and health service assistant
SSH: Social and health service helper
