# Supplementary material for: Lack of focus on nutrition and documentation in nursing homes, home care- and home nursing: the self-perceived views of the primary care workforce
Source: BMC Health Serv Res. 2019 Sep 6;19:642. doi: 10.1186/s12913-019-4450-1 (PMC6729022; doi:10.1186/s12913-019-4450-1)
Supplement: Supplementary file 1 — Interview Guide. (DOCX 17 kb) [file 12913_2019_4450_MOESM1_ESM.docx]

# supplementary file: Interview guide

| ROUTINES  When you hear the word nutrition ANDhome care / home nursing  - what do you think?  Try to tell me a little more about what you understand about nutrition?  Can you give an example of how you work with nutrition?  What are your specific daily tasks or routines in relation to nutrition?  Is nutrition something you think about on a daily basis?  How is it on the agenda daily?  How do you talk about it?  Can you tell what significance nutrition can have for patients?  In the questionnaire that you all filled out regarding your nutrition and documentation routines seem to have a great variety in the answers.That is, there are very different routines in the municipality in relation to whether action plans are prepared in the field of nutrition, systematic actions are taken, documents about it in the journal, etc.  How do you experience it in your daily life? What does this mean for your work?  Now that we talk about continuity or continuous patient care - how do you ensure this happens?  What are your thoughts on this?  In the questionnaires it is mentioned by several that it is difficult to get actions done by the citizen and ensure follow-up.  The lack of communication with the night team, etc.  How do you experience it in your daily life? What does this mean for your work?  These differences in routines, etc. - does it matter to patients?  What significance can it have to the citizen if a course is not coherent or there is no continuity in the process?  Action plans / focus areas in nutrition - What do you think about it and is it something you use?  When do you think it is necessary to develop nutrition action plans?  How can a typical nutrition plan contain or revolve around?  Can you give some examples of when it has been most relevant for you to prepare or have an action plan in nutrition? And what areas did it contain?  How important do you think action plans really are?  (Can you say a little more about it (why / why not?)  Does it matter?  Do you think this is important?  Try to tell me a little more about what you understand by documentation?  Can you tell how you work with documentation in your daily life?  Can you say a little about how you actually document?  Do you have any particular routines for this? | KNOWLEDGE  Now when we talk about nutrition and focus areas, it might be obvious to go back a step and ask:  How do you become aware if a patient is at risk of malnutrition or perhaps malnourished? (Do you use screening instruments or other tools?)  And what do you do then?  Can you tell of a situation where some observations on the patient really made the alarm bells ring?  How do you act on your observations - can you give me some examples?  Try telling me about a situation where you talked about nutrition with the citizen?  Why did you do that and what did you talk about?  How do you ensure follow-up and evaluation?  What do you base your advice and guidance to the citizens on (evidence / experience)  Try to mention some of the last advice you have given your citizens?  If a colleague e.g. have prepared an action plan or created a focus area in nutrition without precise action instructions or it is difficult to follow and understand - what do you do?  (Do you talk to and revise it with that colleague? Fail to use it? Does it even revise without the involvement of the person who drafted it?)  What do you think about feed-back?  Try to say something about how you provide feed-back (sparse with each other) to each other in daily life and especially in terms of nutrition and documentation?  Now that you become aware that the citizen has a nutritional problem - what do you do?  Are you addressing anyone? (what did you do before the dietician came?)  How do you use each other as colleagues to talk to each other about a patient's nutritional status? | ATTITUDES  Do you consider nutrition as part of your area of ​​work?  Is it important? Why is it important? Is it a priority? Isn't that a priority?  Now we have talked about the division of responsibilities in relation to documentation - Can you say something about the division of responsibilities in relation to nutrition?  ORGANIZATION  You have a different educational background - do you have different responsibilities?  Is this division of responsibilities clear to you all?  Is it written down and clarified? Are you introduced to this?  What is your specific responsibility and what other partners or colleagues do you see also have a responsibility and if so what responsibility? Does anyone have the main responsibility for nutrition or is it everyone's responsibility (patient, relatives, colleagues, doctor, dietitian)?  Tell us a little about the importance of the documentation when communicating with your colleagues?  Is this your primary form of communication?  What are the advantages or disadvantages of the documentation?  Can there be something about systematics that ensures you get it all and is a way of communicating with partners?  Does it support your routines and ensure that care and treatment are documented?  Now we have talked about action plans and focus areas - of course, all that is about documenting.  Who is responsible for documentation?  Can you say a little about the division of responsibilities in relation to documenting nutrition (not just drawing up action plans?)  If everyone is in charge - are there any different variations in responsibility?  CONTEXT  In terms of nutrition care and treatment, are there any limitations or benefits to working in the primary sector (in the patient's own home)?  Can you give me an example from your own practice, where you can should "think-out-of-the-box" due to the physical conditions or conditions, to ensure that the patient received the best nutritional care and treatment? |
| --- | --- | --- |
